# Proteomic analysis reveals some common proteins in the kidney stone matrix

**DOI:** 10.7717/peerj.11872

**Published:** 2021-07-27

**Authors:** Yuanyuan Yang, Senyuan Hong, Cong Li, Jiaqiao Zhang, Henglong Hu, Xiaolong Chen, Kehua Jiang, Fa Sun, Qing Wang, Shaogang Wang

**Affiliations:** 1 Department of Urology, Tongji Hospital, Tongji Medical College, Huazhong University of Science and Technology, Wuhan, Hubei, China; 2 Department of Urology, Guizhou Provincial People’s Hospital, Guizhou University, Guiyang, Guizhou, China; 3 Department of Research Laboratory Center, Guizhou Provincial People’s Hospital, Guizhou University, Guiyang, Guizhou, China

**Keywords:** Bioinformatic, Nephrolithiasis, Proteomics, Stone matrix, Biomarker

## Abstract

**Background:**

Proteins are the most abundant component of kidney stone matrices and their presence may reflect the process of the stone’s formation. Many studies have explored the proteomics of urinary stones and crystals. We sought to comprehensively identify the proteins found in kidney stones and to identify new, reliable biomolecules for use in nephrolithiasis research.

**Methods:**

We conducted bioinformatics research in November 2020 on the proteomics of urinary stones and crystals. We used the ClusterProfiler R package to transform proteins into their corresponding genes and Ensembl IDs. In each study we located where proteomic results intersected to determine the 20 most frequently identified stone matrix proteins. We used the Human Protein Atlas to obtain the biological information of the 20 proteins and conducted Gene Ontology (GO) and Kyoto Encyclopedia of Genes and Genome (KEGG) analysis to explore their biological functions. We also performed immunohistochemistry to detect the expression of the top five stone matrix proteins in renal tissue.

**Results:**

We included 19 relevant studies for analysis. We then identified 1,409 proteins in the stone matrix after the duplicates were removed. The 20 most-commonly identified stone matrix proteins were: S100A8, S100A9, uromodulin, albumin, osteopontin, lactotransferrin, vitamin K-dependent protein Z, prothrombin, hemoglobin subunit beta, myeloperoxidase, mannan-binding lectin serine protease 2, lysozyme C, complement C3, serum amyloid *P*-component, cathepsin G, vitronectin, apolipoprotein A-1, eosinophil cationic protein, fibrinogen alpha chain, and apolipoprotein D. GO and KEGG analysis revealed that these proteins were typically engaged in inflammation and immune response.Immunohistochemistry of the top five stone matrix proteins in renal tissue showed that the expression of S100A8, S100A9, and osteopontin increased, while uromodulin decreased in kidney stone patients. Albumin was rarely expressed in the kidney with no significant difference between healthy controls and kidney stone patients.

**Conclusion:**

Proteomic analysis revealed some common inflammation-related proteins in the kidney stone matrix. The role of these proteins in stone formation should be explored for their potential use as diagnostic biomarkers and therapeutic targets for urolithiasis.

## Introduction

Kidney stones are a common public health problem. It is estimated that 10% to 12% of the general population will suffer from nephrolithiasis in their lifetime (Coe, Evan & Worcester, 2005). The prevalence of kidney stones is currently 6.4% of the general population in China and this incidence has increased over the past four decades in Western countries (Zeng et al., 2017; Scales et al., 2012). The 10-year recurrence rate for kidney stones may be as high as 50%, resulting in repetitive treatments and a huge economic burden (Uribarri, Oh & Carroll, 1989; Geraghty et al., 2020). Thus, it is important to explore the pathogenesis of nephrolithiasis and provide a theoretical basis for its treatment and prevention.

Kidney stones are composed of 97–98% mineral salts and 2–3% organic matrix (Boyce, 1968). The organic matrix, including proteins, lipids, glycosaminoglycans, and carbohydrates, plays a role in modulating the formation of stones (Boyce & Garvey, 1956). Proteins are the most abundant component in a kidney stone and comprise approximately 64% of the stone matrix (Boyce, 1968). The identification of the matrix proteins contributes to a better understanding of a stone’s formation. Advancements in proteomic techniques and the introduction of mass spectrometry have improved the study of the proteomics in urinary stones and crystals (Mushtaq et al., 2007; Canales et al., 2008; Merchant et al., 2008; Chen et al., 2008; Canales et al., 2009; Thurgood & Ryall, 2010; Thurgood et al., 2010; Canales et al., 2010; Kaneko et al., 2011; Jou et al., 2012; Kaneko et al., 2012; Okumura et al., 2013; Boonla et al., 2014; Kaneko et al., 2014; Kaneko et al., 2015; Martelli et al., 2016; Witzmann et al., 2016; Kaneko et al., 2018; Wesson et al., 2019). More than one thousand proteins have been detected in the stone matrix to date. Some common proteins, including S100A8, S100A9, and osteopontin (OPN) are frequently detected in stones and may reveal a potentially universal pattern for stone formation.

We conducted a systemic review of studies that focused on the proteomics of the stone matrix to determine the proteins in kidney stones and to identify those that were the most frequently occuring. We performed bioinformatic analysis to explore the function of the top 20 matrix proteins and immunohistochemistry to detect the expression of the top five stone matrix proteins in renal tissue. We sought to provide new and reliable biomolecules for urolithiasis research.

## Methods

### Literature search

We conducted a systematic literature search of Medline, Embase, and the Web of Science databases in November 2020. The following search strategy was used: (((((urin*) OR kidney) OR renal)) AND (((((stone) OR calculi) OR calcium) OR matrix) OR crystal)) AND proteomic. We included studies that were written in English and associated with proteomics of urinary stones and crystals in our study.

### Bioinformatic analysis

We used clusterProfiler package in R version 4.0.0 to transform the proteins to related genes and Ensembl IDs (Yu et al., 2012) to unify the names of proteins in different studies. We took the intersection of the proteomic results from each study and identified the 20 most common stone matrix proteins. We searched the Human Protein Altas to obtain the biological information of the matrix proteins (https://www.proteinatlas.org/) (Uhlén et al., 2015). Gene Ontology (GO) and Kyoto Encyclopedia of Genes and Genome (KEGG) analyses were performed using the OmicShare tool, a free online platform for data analysis (http://www.omicshare.com/tools) to explore the biological function of the 20 proteins. Enrichment results were filtered with a false discovery rate (FDR) of < 0.05.

### Immunohistochemistry

Renal tissue was collected from patients undergoing nephrectomy due to kidney stones or renal tumor. Tissue from kidney stone patients was classified as the stone group and normal paracancer tissue from kidney tumor patients was defined as the control group. The samples were fixed with formalin and embedded in paraffin for routine sectioning. Slices of the tissue were incubated with anti-S100A8 antibody (ab92331; Abcam, Cambridge, UK), anti-S100A9 antibody (ab92507; Abcam, Cambridge, UK), anti-uromodulin antibody (A01303-2; Boster, Selangor, Malaysia), anti-OPN (ab8448; Abcam, Cambridge, UK), and anti-albumin antibody (A1363; Abclonal, Wuhan, China) for immunohistochemistry. We used Image J software version 1.52 (National Institute of Mental Health, Bethesda, MD, USA) was used to quantify the relative area of the positive staining area. The ethical review board of Tongji Hospital, Tongji Medical College, Huazhong University of Science and Technology approved the collection and use of tissue samples (2019S1147). The written form of informed consent was obtained from all patients.

### Statistical analysis

Measurement data are presented as mean ± standard deviations. A Student’s t-test was conducted using Prism 9.0 for statistical analysis. A *p*-value 0.05 was considered to be statistically significant.

## Results

### An overview of the included studies

**Figure 1:**
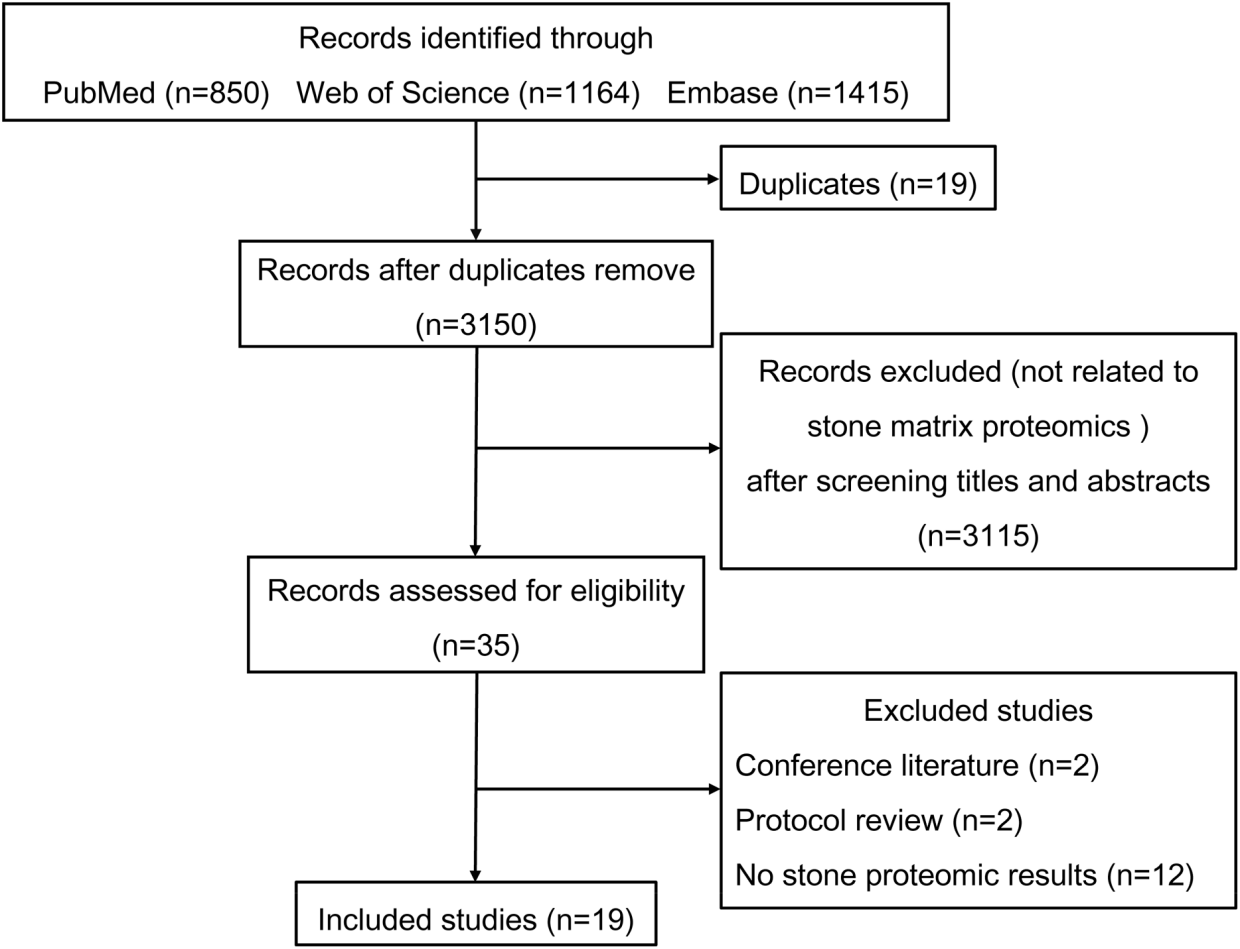
Flowchart of the literature search and study selection.

Figure 1 shows the process used to select studies. We included 19 studies exploring the proteomics of urinary stones and crystals and an overview of these studies is shown in Table 1. Liquid chromatography-tandem mass spectrometry (LC-MS/MS) was the most commonly-used proteomic technique and provided high-throughput amino acid sequence data. Most studies focused on calcium oxalate (CaOx) and uric acid (UA) stones but occasionally rare stone types such as matrix and CaCO3 stones were also studied. Thurgood et al. also reported the protein files in urinary crystals from a healthy population (Thurgood & Ryall, 2010; Thurgood et al., 2010). Many common inflammatory proteins were identified in various studies, indicating the involvement of inflammation in stone formation.

**Table 1:** Basic information of included studies. Detailed information of the 19 studies we included in our research.

Years	Authors	Sample size	Stone composition	Proteomic technique	Verification methods	Proteins identified	Main findings
2007 (Mushtaq et al., 2007)	Mushtaq et al.	40	CaOx	1D PAGE LC-MS/MS	Western blot	4	Myeloperoxidase, α-defensin and calgranulin were identified from inner core of CaOx stones and they promoted the aggregation of CaOx crystals. Osteopontin was detected both in the inner and outer matrix of CaOx stones.
2008 (Canales et al., 2008)	Canales et al.	7	CaOx	LC-MS/MS	NA	68	A significant number of inflammatory proteins, such as immunoglobulin, α-defensin-3, clusterin, complement C3a, kininogen, calgranulin and fibrinogen, were found in CaOx stones matrix.
2008 (Merchant et al., 2008)	Merchant et al.	4	CaOx	LC-MS/MS	Western blot	158	A total of 58 prevalent proteins were detected in at least two of the three LC-MS/MS analyses. Pathway analysis suggested that a significant fraction of CaOx stone matrix proteins participate in inflammatory processes.
2008 (Chen et al., 2008)	Chen et al.	10	CaOx	1D PAGE LC-MS/MS	NA	11	There were abundant proteins with molecular weight around 27, 14, and 10 kDa in CaOx stones matrix. Methylation, deamidation, and oxidation were indentified with mass spectroscopy in these proteins.
2009 (Canales et al., 2009)	Canales et al.	1	Matrix stone	LC-MS/MS	NA	33	Protein file of matrix stones included many similar inflammatory proteins seen in previous proteomic studies of CaOx stone matrix, indicating a primary inflammatory mechanism behind matrix stones.
2010 (Thurgood & Ryall, 2010)	Thurgood et al.**^#^**	5	HA	LC-MS/MS	NA	36	Binding of proteins to urinary hydroxyapatite, brushite, and uric acid crystals is selective and distinct. Several proteins consistently detected in the healthy urine crystal extracts, such as osteopontin, prothrombin and S100A9, have been previously implicated in kidney stone disease.
			Brushite	LC-MS/MS	NA	65	
			UA	LC-MS/MS	NA	7	
2010 (Thurgood et al., 2010)	Thurgood et al.**^#^**	5	COM	LC-MS/MS	2D SDS-PAGE	14	The incorporation of proteins into COM and COD crystals from healthy human urine was selective. Principal proteins in COM crystal extracts were prothrombin fragment 1, S100A9, and IGκV1-5, while those in COD crystals included osteopontin, IGκV1-5, S100A9, annexin A1, HMW kininogen-1, and inter-α-inhibitor.
			COD	LC-MS/MS	NA	34	
2010 (Canales et al., 2010)	Canales et al.	13	CaOx	LC-MS/MS	NA	49	CaOx and CaP stones shared similar matrix proteins associated with inflammatory response, indicating that inflammation play an important role in calcium stone formation, no matter as an origin role or a secondary response.
		12	CaP	LC-MS/MS	NA	45	
2011 (Kaneko et al., 2011)	Kaneko et al.	1	UA, COM	LC-MS/MS	NA	32	Calcium-binding proteins, such as calprotectin, psoriasin, calprotectin and so on, were identified in stones from patients with hyperuricemia. They may play a significant role in the formation of kidney uric acid stones.
2012 (Jou et al., 2012)	Jou et al.	5	UA	LC-MS/MS	Western blot	242	The function of proteins identified from uric acid stones is mainly engaged in inflammatory process and lipid metabolism, implying a possible relation between lipotoxicity and stone formation.
2012 (Kaneko et al., 2012)	Kaneko et al.	17	CaOx, UA	1D PAGE LC-MS/MS	Western blot	30	Uromodulin and albumin are often detected in stones. Osteopontin, prothrombin, protein S and protein Z are identified specifically in calcium oxalate stones. Immunoglobin G fragments are detected in uric acid stones.
2013 (Okumura et al., 2013)	Okumura et al.	9	CaOx	LC-MS/MS	Western blot	92	Prothrombin, osteopontin, S100A8 and S100A9 were found in most stones, some samples had high contents of prothrombin and osteopontin, while others had high contents of calgranulins and neutrophil-enriched proteins.
2014 (Boonla et al., 2014)	Boonla et al.	16	COM, UA, MAP	1D PAGE LC-MS/MS	Western blot	62	Kidney stones greatly contained inflammatory and fibrotic proteins, indicating that inflammation and fibrosis are involved in the formation of stones. S100A8 and fibronectin were the most abundant protein in stone matrix.
2014 (Kaneko et al., 2014)	Kaneko et al.	1	CaCO3, CaOx	1D PAGE LC-MS/MS	NA	53	Matrix proteins from calcium carbonate stone are mostly associated with cell adhesion and cytoskeleton. These identified proteins may play an important role on urolithiasis in alkaline condition.
2015 (Kaneko et al., 2015)	Kaneko et al.	16	COM, COD, HA	1D PAGE LC-MS/MS	NA	65	Many plasma proteins were frequently detected in stone matrix regardless of the stone components. Identified proteins were involved in inflammation, coagulation process, and osteometabolism.
2016 (Martelli et al., 2016)	Martelli et al.	4	Matrix stone	1D PAGE LC-MS/MS	NA	142	S100A8, S100A9 and neutrophil defensin were identified as the main component of matrix stones. Inflammatory process may be the origin of this kind of rare soft calculi formation but not be the consequence.
2016 (Witzmann et al., 2016)	Witzmann et al.	2	CaOx	LC-MS/MS	NA	1059	A more complex stone matrix proteome than previously studies was reported. Matrix proteins were related to immune response, inflammation, injury, and tissue repair.
2018 (Kaneko et al., 2018)	Kaneko et al.	1	COM, UA	1D PAGE LC-MS/MS	NA	59	Proteins relevant to cell adhesion, self-defense, and plasma commonly play a major role in the generation of stone. The proteins in the interface likely function to enlarge the stone *via* the addition of different crystals.
2019 (Wesson et al., 2019)	Wesson et al.	8	CaOx	LC-MS/MS	NA	366	Osteopontin, mannan-binding lectin serine protease 2, vitamin K-dependent protein Z, prothrombin, and hemoglobin β chain were prominently enriched in matrix, accounting for a mass fraction of >30% of matrix protein. Many identified matrix proteins are reported in intracellular or nuclear locations, indicating a significant role of cell injury in stone formation.

**Notes:**

# Crystals are isolated from the urine of healthy people without urinary stones.

COM, Calcium oxalate monohydrate; COD, Calcium oxalate dihydrate; CaCO3, Calcium carbonate; CaOx, Calcium oxalate; CaP, Calcium phosphate; UA, Uric acid; MAP, Magnesium ammonium phosphate; HA, Hydroxyapatite.

### Identifying the 20 most common proteins in stone matrices

**Figure 2:**
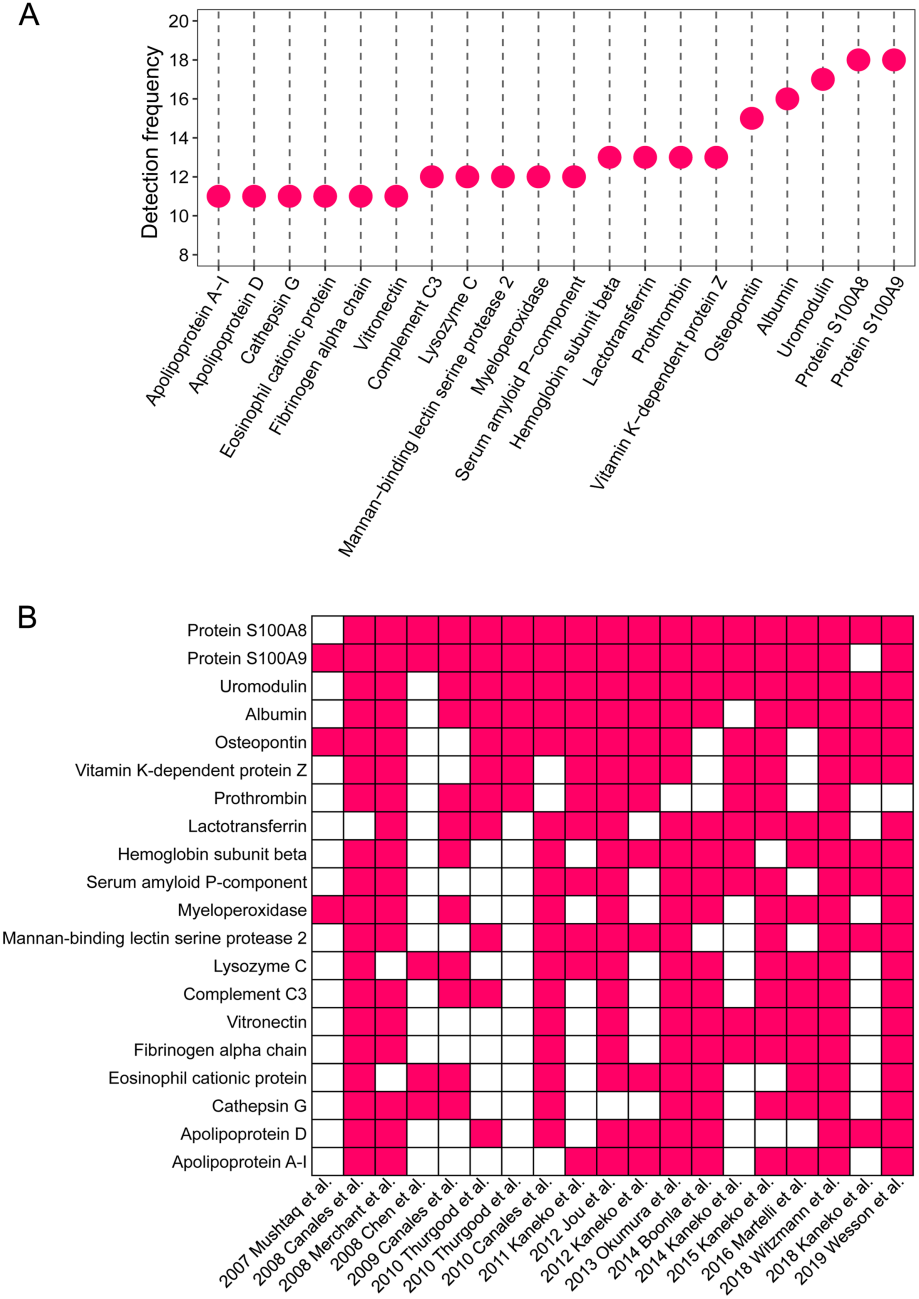
The 20 most common proteins in stone matrix. (A) Detected frequency of the top 20 proteins in stone matrix. (B) Detection of the top 20 proteins in each study.

The detailed proteomic results of each study are presented in File 1. A total of 1,409 proteins were detected in the stone matrix after removing duplicates (File 1). We intersected each study to identify the most common stone matrix proteins. The most frequently detected proteins in the stone matrix were: S100A8, S100A9, uromodulin, albumin, OPN, lactotransferrin, vitamin K-dependent protein Z, prothrombin, hemoglobin subunit beta, myeloperoxidase, mannan-binding lectin serine protease 2, lysozyme C, complement C3, serum amyloid *P*-component, cathepsin G, vitronectin, apolipoprotein A-1, eosinophil cationic protein, fibrinogen alpha chain, and apolipoprotein D. Figure 2 shows the exact detection frequency of each protein. We also conducted a subgroup analysis of the studies and focused only on CaOx stones. We found that the 20 most common proteins in CaOx stone matrices were same as the above 20 proteins (File 2). We searched the online Human Protein Altas database to explore the biology of these proteins. Uromodulin, OPN, lysozyme C, and apolipoprotein D had medium to high expression in renal tubular epithelial cells, while the remaining proteins were rich in other tissues (Table 2).

**Table 2:** Biological information of the 20 most common proteins in stone matrix. Biological information of the 20 most common proteins in stone matrix (from the Human Protein Altas).

Proteins	GENE	Tissue specificity	Blood specificity	Expression in glomeruli	Expression in renal tubules	Biological process
S100A8	S100A8	Blood, bone marrow, esophagus, tongue	Neutrophil, classical monocyte	Rare	Rare	Apoptosis, autophagy, chemotaxis, immunity, inflammatory response, innate immunity
S100A9	S100A9	Blood, bone marrow, esophagus, tongue	Neutrophil, classical monocyte	Rare	Rare	Apoptosis, autophagy, chemotaxis, immunity, inflammatory response, innate immunity
Uromodulin	UMOD	Kidney	None	Rare	High	Ciliopathy, disease mutation, nephronophthisis
Albumin	ALB	Liver	Naive CD4 T-cell	Rare	Rare	Cancer-related genes, disease mutation
Osteopontin	SPP1	Gallbladder, kidney, placenta	Neutrophil	Rare	High	Biomineralization, cell adhesion
Lactotransferrin	LTF	Bone marrow, salivary gland, seminal vesicle	Non-classical monocyte, neutrophil	Rare	Rare	Immunity, ion transport, osteogenesis, iron transport, transcription, transcription regulation, transport
Vitamin K-dependent protein Z	PROZ	Liver	Intermediate monocyte, T-reg	Rare	Rare	Blood coagulation, hemostasis
Prothrombin	F2	Liver	None	Rare	Rare	Acute phase, blood coagulation, hemostasis
Hemoglobin subunit beta	HBB	Bone marrow	Neutrophil, plasmacytoid DC	Rare	Rare	Oxygen transport, transport
Myeloperoxidase	MPO	Bone marrow	Classical monocyte, intermediate monocyte, myeloid DC, neutrophil	Rare	Rare	Hydrogen peroxide
Mannan-binding lectin serine protease 2	MASP2	Liver	Low immune cell specificity	Rare	Rare	Complement pathway, immunity, innate immunity
Lysozyme C	LYZ	Blood, salivary gland	Classical monocyte, myeloid DC	Rare	Medium	Amyloidosis, disease mutation
Complement C3	C3	Liver	Non-classical monocyte	Rare	Rare	Complement alternate pathway, complement pathway, fatty acid metabolism, host-virus interaction, immunity, inflammatory response, Innate immunity, lipid metabolism
Serum amyloid P-component	APCS	Liver	None	Rare	Rare	Calcium, lectin, metal-binding
Cathepsin G	CTSG	Bone marrow	Neutrophil, classical monocyte, plasmacytoid DC, NK-cell, myeloid DC, memory CD8 T-cell, naive CD8 T-cell	Rare	Rare	Antibiotic, antimicrobial, hydrolase, protease, serine protease
Vitronectin	VTN	Liver	Naive CD8 T-cell	Rare	Rare	Cell adhesion
Apolipoprotein A-1	APOA1	Liver	Plasmacytoid DC	Rare	Rare	Cholesterol metabolism, lipid metabolism, lipid transport, steroid metabolism, transport, sterol metabolism
Eosinophil cationic protein	RNASE3	Blood, bone marrow	Eosinophil	Rare	Rare	Antibiotic, antimicrobial, endonuclease, hydrolase, nuclease
Fibrinogen alpha chain	FGA	Liver	None	Rare	Rare	Adaptive immunity, blood coagulation, hemostasis, immunity, innate immunity
Apolipoprotein D	APOD	Breast	Memory B-cell	Rare	High	Transport

### Biological function of the 20 most common proteins in stone matrices

**Figure 3:**
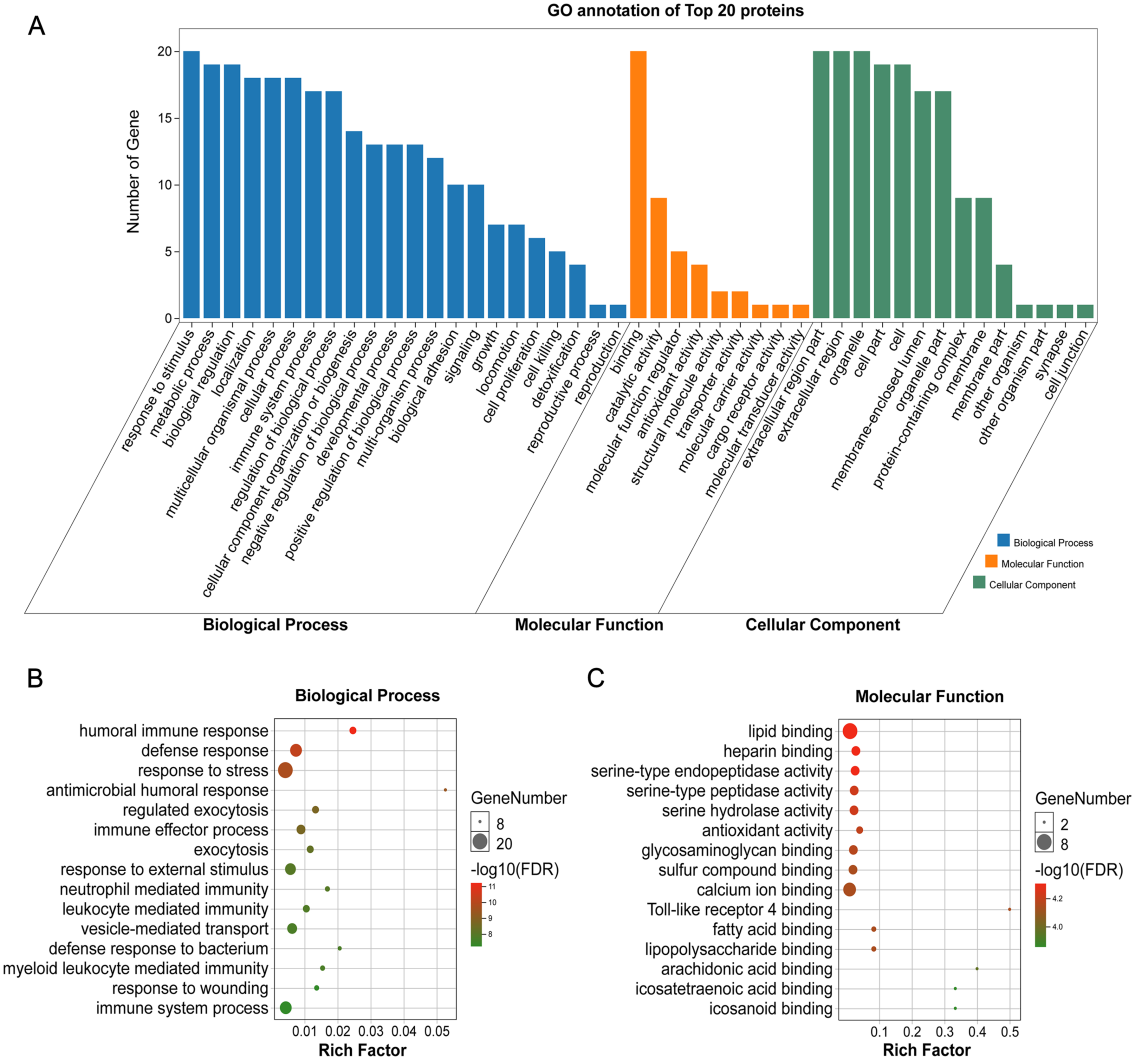
GO analysis of the 20 most common proteins in stone matrix. (A) GO annotation. (B) GO biological process enrichment analysis. ****(C) GO molecular function enrichment analysis. Rich Factor referred to the ratio of the number of enriched genes in the GO category to the total genes in that category. FDR referred to false discovery rate. FDR < 0.05 was set as the cut-off value.

**Figure 4:**
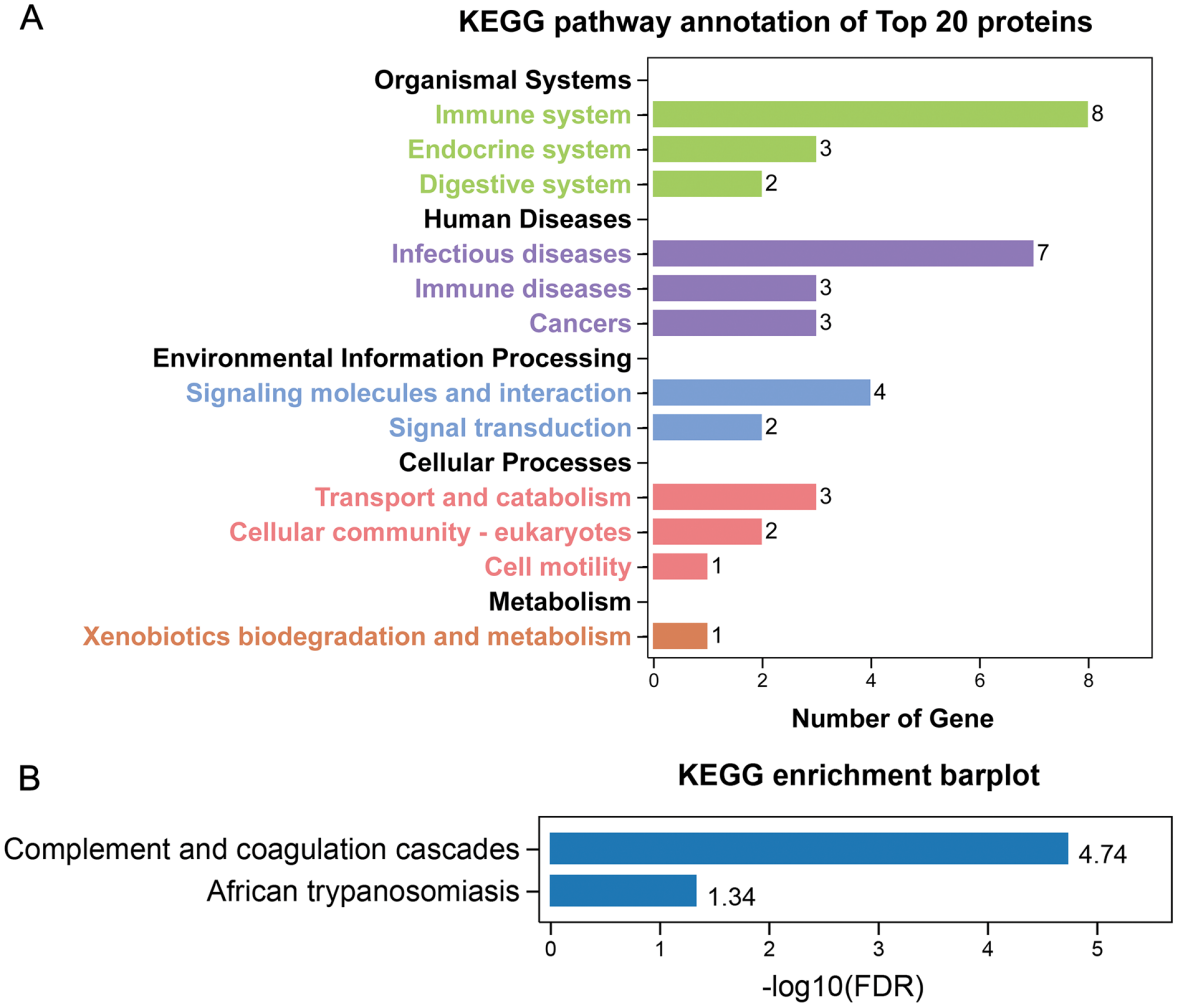
KEGG analysis of the 20 most common proteins in stone matrix. (A) KEGG annotation. (B) KEGG disease enrichment analysis.

GO annotation showed that the top 20 proteins were involved in the stimulus response, binding activity, and extracellular region. They played a role in the immune response, indicating that stone formation was associated with the inflammatory response. These 20 proteins showed a strong ability to bind with calcium ions, which may explain why they appeared in the stone matrix (Fig. 3). KEGG annotation also showed that the top 20 proteins participated in immune and infectious disease responses. They were enriched in the complement and coagulation cascades pathway, which activates the complements involved in the immune response and coagulation cascades associated with inflammation (Fig. 4).

### The expression of the top five stone matrix proteins in renal tissue

**Figure 5:**
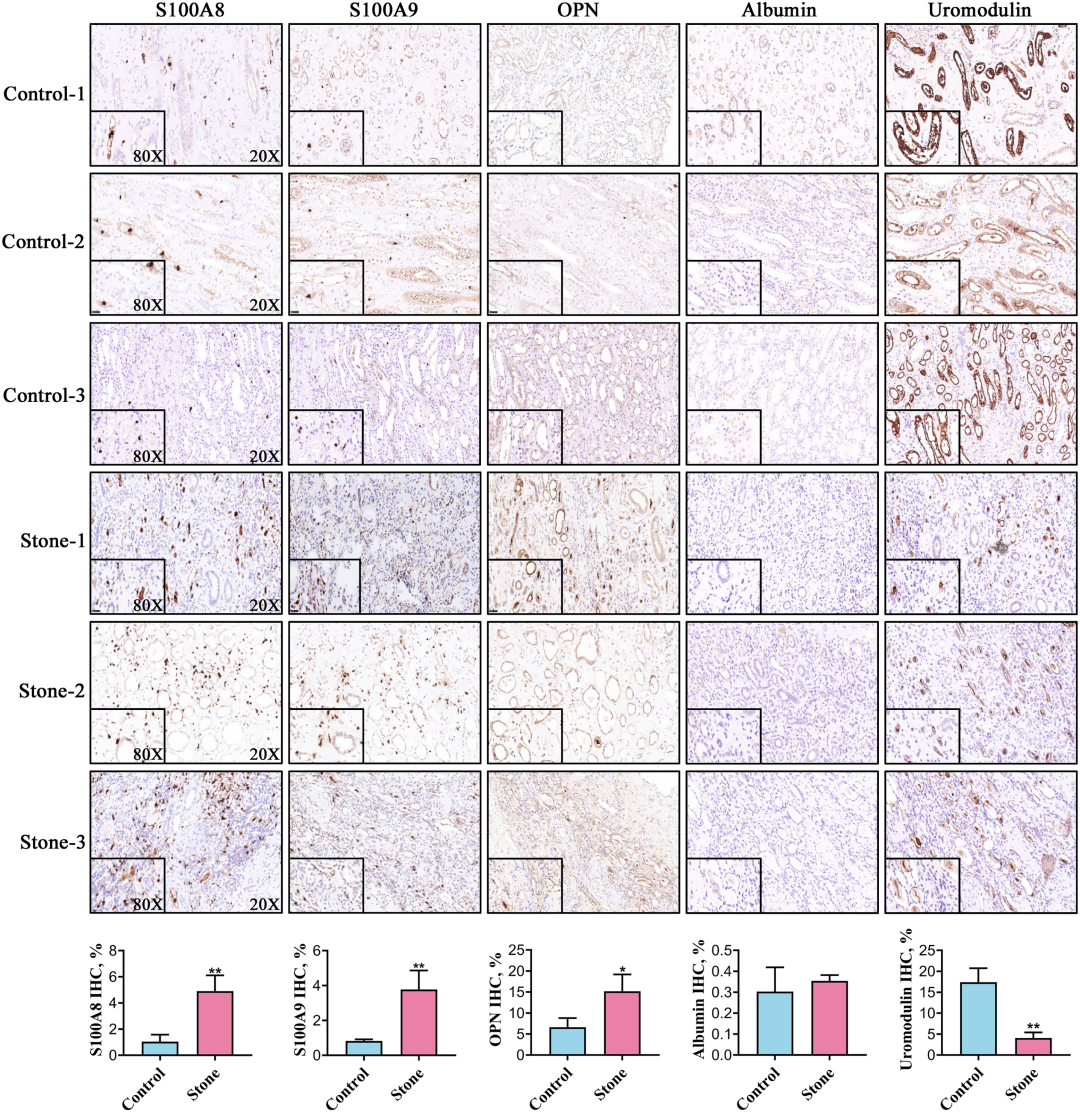
Representative images for detection of the top five stone matrix proteins in renal tissue. The renal expression of S100A8, S100A9, and osteopontin were increased, while uromodulin was decreased in kidney stone patients. Albumin was rarely expressed in kidney and there was no significant difference between healthy controls and kidney stone patients; *P < 0.05 vs Control; **P < 0.01 vs Control.

Among the 20 common stone matrix proteins, S100A8 and S100A9 were the most frequently detected and they appeared in all kinds of urinary stones. Our previous study demonstrated that urinary exosomes from kidney stone patients were rich in S100A8 and S100A9 (Wang et al., 2020). Immunohistochemistry results showed that the expression of S100A8, S100A9 and OPN was significantly increased in renal tissue from kidney stone patients. In contrast, the expression of uromodulin was decreased in the renal tissue of kidney stone patients. Positive S100A8 and S100A9 staining were restricted in cells within vessels in normal kidney tissues. However, S100A8 and S100A9 were mainly expressed in the renal interstitium of kidney stone patients. The expression of OPN and uromodulin typically occured in renal tubular epithelial cells in both the control and stone groups. Albumin was rarely detected in the kidney and there was no significant difference between healthy controls and kidney stone patients (Fig. 5).

## Discussion

Stone matrix proteins play a role in modulating the nucleation, aggregation and growth of urinary crystals, which may affect stone formation (Narula et al., 2020). Several investigators in the last 20^th^ century attempted to identify the proteins in stones, but were limited by the lack of high throughput technology (Binette et al., 1996; Sugimoto et al., 1985; Jones & Resnick, 1990). Thousands of proteins have been identified in the stone matrix with the introduction of mass spectrometry, which has improved the understanding of the pathogenesis of urolithiasis.

The binding of proteins to various stones is selective despite the presence of some common proteins. We identified the 20 most common proteins in the stone matrix. These proteins typically engaged in immune and inflammatory responses, indicating the role of inflammation in stone formation. Recent studies have reported that the macrophage-related immune response participates in stone formation and immunotherapy may be used to treat kidney stone disease (Dominguez-Gutierrez et al., 2020). Only four of the 20 most common matrix proteins showed medium-to-high expression in the kidney (Table 2). Other non-renal-specific proteins were throught to originate from plasma proteins physiologically filtered through the glomeruli, products of immune cells infiltrated in the renal interstitium, or plasma proteins pathologically exudated due to the injury and infection caused by stones.

S100A8 and S100A9 were detected with the highest frequency from the top five matrix proteins. These proteins belong to the S100 calcium binding family and are primarily derived from neutrophils, monocytes and M1 macrophages (Pruenster et al., 2016; Dessing et al., 2015). As critical alarmin, S100A8 and S100A9 play an important role in regulating the immune response. They mediate the production of proinflammatory cytokines and the recruitment of leukocytes (Wang et al., 2018). New et al. (2013) reported that S100A9-rich vesicles from macrophages have a powerful potential for calcification in 2013. We previously demonstrated that the expression of urinary exosomal S100A8 and S100A9 in kidney stone patients was higher than in healthy controls (Wang et al., 2020). In this study, the expression of S100A8 and S100A9 in renal tissue was also found to be elevated in kidney stone patients. In addition, S100A8 and S100A9 were detected in the renal interstitium of kidney stone patients, which may originate from macrophages in the kidney. The potential role of S100A8 and S100A9 in stone formation is worthy of further exploration. They may be valuable as both diagnostic biomarkers and therapeutic targets for urolithiasis.

Uromodulin, also known as Tamm-Horsfall protein (THP), is the third most common stone matrix protein. It is secreted from the thick ascending limb of Henle’s loop (Gokhale, Glenton & Khan, 2001). Previous *in vitro* studies have shown that THP could inhibit the aggregation of calcium oxalate and calcium phosphate crystals (Kumar & Lieske, 2006). Liu et al. (2010) identified crystals deposited in the kidneys of THP knockout mice at as early as two months of age. Transmission electron microscopy showed that the deposits were spherical in shape with multiple layers, which is similar to human calcium oxalate stones (Liu et al., 2010). THP is thought to play an inhibitory role in stone formation. However, urinary THP excretion decreases in kidney stone patients (Lau et al., 2008). We also found that the expression of uromodulin decreased in the renal tissue of kidney stone patients. Stone formers have been reported to excrete defective THP, which lacks sialic acid and reducing its effectiveness in inhibiting stone formation (Knörle et al., 1994; Viswanathan et al., 2011). Increasing the expression of THP and restoring its function may be effective in the prevention of kidney stones.

Albumin is the fourth most common stone matrix protein and it is rarely expressed in the kidney. The albumin in the stone matrix was thought to originate from plasma proteins filtered through the glomeruli. Albumin is reported to be a powerful nucleator of COD and the polymer form is more active than the monomer form (Cerini et al., 1999). Cerini et al. (1999) thought that promotion of COD crystallization by albumin of crystallization mayight be a protective factor for urine stability, because the with rapid nucleation of small crystals caused the, the saturation levels to fall, preventing COM formation and aggregation with subsequent stone formation be prevented (Cerini et al., 1999).

OPN, the fifth most common stone matrix protein, is expressed in the distal renal tubules and thick ascending limbs of Henle’s loop (Xie et al., 2001). The expression of OPN has been shown to increase in the kidneys of stone patients and experimental models, which was validated by our findings (Kleinman, Wesson & Hughes, 2004). The role of OPN in stone formation remains controversial and may depend on its phosphorylation level (Wang et al., 2008). Wesson et al. (2003) reported that OPN knockout mice developed more CaOx crystals in their kidney than wild type mice in the hyperoxaluria model. Their previous study reported that OPN favors the formation of calcium oxalate dihydrate (COD) over calcium oxalate monohydrate (COM). COD is less adherent to renal epithelial cells than COM, which may partly explain the antilithiatic effect of OPN (Wesson et al., 1998). However, other studies have shown that OPN may increase the risk of stone formation by promoting crystal adherence to the renal epithelium (Yamate et al., 1996; Yamate et al., 1999).

## Conclusions

We identified some common inflammation-related proteins that play a role in stone formation based on proteomic data, to help determine the pathogenesis of human urolithiasis. However, the mere detection of a protein does not explain how it participates in stone formation. Future studies are needed to identify the role of these proteins in stone formation and are expected to provide new diagnostic biomarkers and therapeutic targets for urolithiasis.

## Supplemental Information

10.7717/peerj.11872/supp-1 Supplemental Information 1 The detailed proteomic results of each study.

10.7717/peerj.11872/supp-2 Supplemental Information 2 Demographic and clinical data of the included subjects and the 20 most common proteins in calcium oxalate stone matrix.

## List of Abbreviations

COM: Calcium oxalate monohydrate
COD: Calcium oxalate dihydrate
CaCO3: Calcium carbonate
CaOx: Calcium oxalate
CaP: Calcium phosphate
FDR: False discovery rate
GO: Gene ontology
HA: Hydroxyapatite
KEGG: Kyoto Encyclopedia of Genes and Genome
LC-MS/MS: Liquid chromatography-tandem mass spectrometry
MAP: Magnesium ammonium phosphate
OPN: Osteopontin
THP: Tamm-Horsfall protein
UA: Uric acid

## Additional Information and Declarations

### Data Availability

The following information was supplied regarding data availability: The detailed proteomic results of each study focusing on proteomics of kidney stones and the demographic characteristic of included patients and a subgroup analysis of the studies and focused only on CaOx stones are available in the Supplementary Files.

## References

[ref-1] Binette JP, Binette MB, Gawinowicz MA, Kendrick N (1996). Urinary stone proteins: an update. Scanning Microscopy.

[ref-2] Boonla C, Tosukhowong P, Spittau B, Schlosser A, Pimratana C, Krieglstein K (2014). Inflammatory and fibrotic proteins proteomically identified as key protein constituents in urine and stone matrix of patients with kidney calculi. Clinica Chimica Acta.

[ref-3] Boyce WH (1968). Organic matrix of human urinary concretions. American Journal of Medicine.

[ref-4] Boyce WH, Garvey FK (1956). The amount and nature of the organic matrix in urinary calculi: a review. Journal of Urology.

[ref-5] Canales BK, Anderson L, Higgins L, Ensrud-Bowlin K, Roberts KP, Wu BL, Kim IW, Monga M (2010). Proteome of human calcium kidney stones. Urology.

[ref-6] Canales BK, Anderson L, Higgins LA, Frethem C, Ressler A, Kim IW, Monga M (2009). Proteomic analysis of a matrix stone: a case report. Urological Research.

[ref-7] Canales BK, Anderson L, Higgins L, Slaton J, Roberts KP, Liu N, Monga M (2008). Comprehensive proteomic analysis of human calcium oxalate monohydrate kidney stone matrix. Journal of Endourology.

[ref-8] Cerini C, Geider S, Dussol B, Hennequin C, Daudon M, Veesler Séphane, Nitsche S, Boistelle R, Berthézène P, Dupuy P, Vazi A, Berland Y, Dagorn J-C, Verdier J-M (1999). Nucleation of calcium oxalate crystals by albumin: involvement in the prevention of stone formation. Kidney International.

[ref-9] Chen WC, Lai CC, Tsai YH, Lin WY, Tsai FJ (2008). Mass spectroscopic characteristics of low molecular weight proteins extracted from calcium oxalate stones: preliminary study. Journal of Clinical Laboratory Analysis.

[ref-10] Coe FL, Evan A, Worcester E (2005). Kidney stone disease. Journal of Clinical Investigation.

[ref-11] Dessing MC, Tammaro A, Pulskens WP, Teske GJ, Butter LM, Claessen N, van Eijk M, van der Poll T, Vogl T, Roth J, Florquin S, Leemans JC (2015). The calcium-binding protein complex S100A8/A9 has a crucial role in controlling macrophage-mediated renal repair following ischemia/reperfusion. Kidney International.

[ref-12] Dominguez-Gutierrez PR, Kwenda EP, Khan SR, Canales BK (2020). Immunotherapy for stone disease. Current Opinion in Urology.

[ref-13] Geraghty RM, Cook P, Walker V, Somani BK (2020). Evaluation of the economic burden of kidney stone disease in the UK: a retrospective cohort study with a mean follow-up of 19 years. BJU International.

[ref-14] Gokhale JA, Glenton PA, Khan SR (2001). Characterization of Tamm–Horsfall protein in a rat nephrolithiasis model. Journal of Urology.

[ref-15] Jones WT, Resnick MI (1990). The characterization of soluble matrix proteins in selected human renal calculi using two-dimensional polyacrylamide gel electrophoresis. Journal of Urology.

[ref-16] Jou YC, Fang CY, Chen SY, Chen FH, Cheng MC, Shen CH, Liao L-W, Tsai Y-S (2012). Proteomic study of renal uric acid stone. Urology.

[ref-17] Kaneko K, Kabeya M, Kondo H, Fukuuchi T, Yamaoka N, Yasuda M, Yamaguchi S (2018). Proteomic analysis of a urinary stone with two layers composed of calcium oxalate monohydrate and uric acid. Nucleosides Nucleotides Nucleic Acids.

[ref-18] Kaneko K, Kobayashi R, Yasuda M, Izumi Y, Yamanobe T, Shimizu T (2012). Comparison of matrix proteins in different types of urinary stone by proteomic analysis using liquid chromatography-tandem mass spectrometry. International Journal of Urology.

[ref-19] Kaneko K, Matsuta Y, Moriyama M, Yasuda M, Chishima N, Yamaoka N, Fukuuchi T, Miyazawa K, Suzuki K (2014). Proteomic analysis of a rare urinary stone composed of calcium carbonate and calcium oxalate dihydrate: a case report. International Journal of Urology.

[ref-20] Kaneko K, Nishii S, Izumi Y, Yasuda M, Yamanobe T, Fukuuchi T, Yamaoka N, Horie S (2015). Proteomic analysis after sequential extraction of matrix proteins in urinary stones composed of calcium oxalate monohydrate and calcium oxalate dihydrate. Analytical Sciences.

[ref-21] Kaneko K, Yoshida N, Okazaki K, Yamanobe T, Yamaoka N, Yasuda M, Ogata N, Yamada Y, Uchida S, Fujimori S (2011). Urinary stone analysis in a patient with hyperuricemia to determine the mechanism of stone formation. Nucleosides Nucleotides Nucleic Acids.

[ref-22] Kleinman JG, Wesson JA, Hughes J (2004). Osteopontin and calcium stone formation. Nephron Physiology.

[ref-23] Knörle R, Schnierle P, Koch A, Buchholz NP, Hering F, Seiler H, Ackermann T, Rutishauser G (1994). Tamm-Horsfall glycoprotein: role in inhibition and promotion of renal calcium oxalate stone formation studied with Fourier-Transform Infrared spectroscopy. Clinical Chemistry.

[ref-24] Kumar V, Lieske JC (2006). Protein regulation of intrarenal crystallization. Current Opinion in Nephrology and Hypertension.

[ref-25] Lau WH, Leong WS, Ismail Z, Gam LH (2008). Qualification and application of an ELISA for the determination of Tamm Horsfall protein (THP) in human urine and its use for screening of kidney stone disease. International Journal of Biological Sciences.

[ref-26] Liu YL, Mo L, Goldfarb DS, Evan AP, Liang F, Khan SR, Lieske JC, Wu X-R (2010). Progressive renal papillary calcification and ureteral stone formation in mice deficient for Tamm-Horsfall protein. American Journal of Physiology-Renal Physiology.

[ref-27] Martelli C, Marzano V, Iavarone F, Huang L, Vincenzoni F, Desiderio C, Messana I, Beltrami P, Zattoni F, Ferraro PM, Buchholz N, Locci G, Faa G, Castagnola M, Gambaro G (2016). Characterization of the protein components of matrix stones sheds light on S100-A8 and S100-A9 relevance in the inflammatory pathogenesis of these rare renal calculi. Journal of Urology.

[ref-28] Merchant ML, Cummins TD, Wilkey DW, Salyer SA, Powell DW, Klein JB, Lederer ED (2008). Proteomic analysis of renal calculi indicates an important role for inflammatory processes in calcium stone formation. American Journal of Physiology-Renal Physiology.

[ref-29] Mushtaq S, Siddiqui AA, Naqvi ZA, Rattani A, Talati J, Palmberg C, Shafqat J (2007). Identification of myeloperoxidase, α-defensin and calgranulin in calcium oxalate renal stones. Clinica Chimica Acta.

[ref-30] Narula S, Tandon S, Kumar D, Varshney S, Adlakha K, Sengupta S, Singh SK, Tandon C (2020). Human kidney stone matrix proteins alleviate hyperoxaluria induced renal stress by targeting cell-crystal interactions. Life Science Part 1 Physiology & Pharmacology.

[ref-31] New SEP, Goettsch C, Aikawa M, Marchini JF, Shibasaki M, Yabusaki K, Libby P, Shanahan CM, Croce K, Aikawa E (2013). Macrophage derived matrix vesicles: an alternative novel mechanism for micro calcification in atherosclerotic plaques. Circulation Research.

[ref-32] Okumura N, Tsujihata M, Momohara C, Yoshioka I, Suto K, Nonomura N, Okuyama A, Takao T (2013). Diversity in protein profiles of individual calcium oxalate kidney stones. PLOS ONE.

[ref-33] Pruenster M, Vogl T, Roth J, Sperandio M (2016). S100A8/A9: from basic science to clinical application. Pharmacology & Therapeutics.

[ref-34] Scales CD, Smith AC, Hanley JM, Saigal CS (2012). Urologic diseases in america project. Prevalence of kidney stones in the United States. European Urology.

[ref-35] Sugimoto T, Funae Y, Rübben H, Nishio S, Hautmann R, Lutzeyer W (1985). Resolution of proteins in the kidney stone matrix using high-performance liquid chromatography. European Urology.

[ref-36] Thurgood LA, Ryall RL (2010). Proteomic analysis of proteins selectively associated with hydroxyapatite, brushite, and uric acid crystals precipitated from human urine. Journal of Proteome Research.

[ref-37] Thurgood LA, Wang T, Chataway TK, Ryall RL (2010). Comparison of the specific incorporation of intracrystalline proteins into urinary calcium oxalate monohydrate and dihydrate crystals. Journal of Proteome Research.

[ref-38] Uhlén M, Fagerberg L, Hallström BM, Lindskog C, Oksvold P, Mardinoglu A, Sivertsson Å, Kampf C, Sjöstedt E, Asplund A, Olsson I, Edlund K, Lundberg E, Navani S, Szigyarto CA, Odeberg J, Djureinovic D, Takanen JO, Hober S, Alm T, Edqvist P-H, Berling H, Tegel H, Mulder J, Rockberg J, Nilsson P, Schwenk JM, Hamsten M, von Feilitzen K, Forsberg M, Persson L, Johansson F, Zwahlen M, von Heijne G, Nielsen J, Pontén F (2015). Tissue-based map of the human proteome. Science.

[ref-39] Uribarri J, Oh MS, Carroll HJ (1989). The first kidney stone. Annals of Internal Medicine.

[ref-40] Viswanathan R, Rimer JD, Kolbach AM, Ward MD, Kleinman JG, Wesson JA (2011). Calcium oxalate monohydrate aggregation induced by aggregation of desialylated Tamm–Horsfall protein. Urological Research.

[ref-41] Wang L, Guan X, Tang R, Hoyer JR, Wierzbicki A, De Yoreo JJ, Nancollas GH (2008). Phosphorylation of osteopontin is required for inhibition of calcium oxalate crystallization. Journal of Physical Chemistry B.

[ref-42] Wang S, Song R, Wang Z, Jing Z, Wang S, Ma J (2018). S100A8/A9 in inflammation. Frontiers in Immunology.

[ref-43] Wang Q, Sun Y, Yang YY, Li C, Zhang JQ, Wang SG (2020). Quantitative proteomic analysis of urinary exosomes in kidney stone patients. Translational Andrology and Urology.

[ref-53] Wesson JA, Worcester EM, Wiessner JH, Mandel NS, Kleinman JG (1998). Control of calcium oxalate crystal structure and cell adherence by urinary macromolecules. Kidney International.

[ref-45] Wesson JA, Johnson RJ, Mazzali M, Beshensky AM, Stietz S, Giachelli C, Liaw L, Alpers CE, Couser WG, Kleinman JG, Hughes J (2003). Osteopontin is a critical inhibitor of calcium oxalate crystal formation and retention in renal tubules. Journal of The American Society of Nephrology.

[ref-46] Wesson JA, Kolbach-Mandel AM, Hoffmann BR, Davis C, Mandel NS (2019). Selective protein enrichment in calcium oxalate stone matrix: a window to pathogenesis?. Urolithiasis.

[ref-47] Witzmann FA, Evan AP, Coe FL, Worcester EM, Lingeman JE, Williams JC (2016). Label-free proteomic methodology for the analysis of human kidney stone matrix composition. Proteome Science.

[ref-48] Xie Y, Sakatsume M, Nishi S, Narita I, Arakawa M, Gejyo F (2001). Expression, roles, receptors, and regulation of osteopontin in the kidney. Kidney International.

[ref-49] Yamate T, Kohri K, Umekawa T, Amasaki N, Isikawa Y, Iguchi M, Kurita T (1996). The effect of osteopontin on the adhesion of calcium oxalate crystals to Madin–Darby canine kidney cells. European Urology.

[ref-50] Yamate T, Kohri K, Umekawa T, Konya E, Ishikawa Y, Iguchi M, Kurita T (1999). Interaction between osteopontin on madin darby canine kidney cell membrane and calcium oxalate crystal. Urologia Internationalis.

[ref-51] Yu GC, Wang LG, Han YY, He QY (2012). ClusterProfiler: an R package for comparing biological themes among gene clusters. Omics-a Journal of Integrative Biology.

[ref-52] Zeng G, Mai Z, Xia S, Wang Z, Zhang K, Wang L, Long Y, Ma J, Li Y, Wan SP, Wu W, Liu Y, Cui Z, Zhao Z, Qin J, Zeng T, Liu Y, Duan X, Mai X, Yang Z, Kong Z, Zhang T, Cai C, Shao Y, Yue Z, Li S, Ding J, Tang S, Ye Z (2017). Prevalence of kidney stones in China: an ultrasonography based cross-sectional study. BJU International.

