# Supplementary material for: Proteomic analysis reveals some common proteins in the kidney stone matrix
Source: PeerJ. 2021 Jul 27;9:e11872. doi: 10.7717/peerj.11872 (PMC8323604; doi:10.7717/peerj.11872)
Supplement: Supplemental Information 2 [file peerj-09-11872-s002.pdf]

Table S1. Demographic and clinical data of the included subjects						
**ID**	**Age (y)**	**Gender**	**Stone type**	**Renal clear cell carcinoma**	**Hypertension**	**Diabetes**
Control-1	47	Male	-	Yes	No	No
Control-2	43	Male	-	Yes	Yes	No
Control-3	57	Male	-	Yes	No	No
Stone-1	53	Male	Calcium oxalate monohydrate/carbonate apatite phosphate	No	No	No
Stone-2	50	Male	Calcium oxalate monohydrate/carbonate apatite phosphate	No	No	No
Stone-3	45	Female	Calcium oxalate monohydrate/carbonate apatite phosphate	No	Yes	No

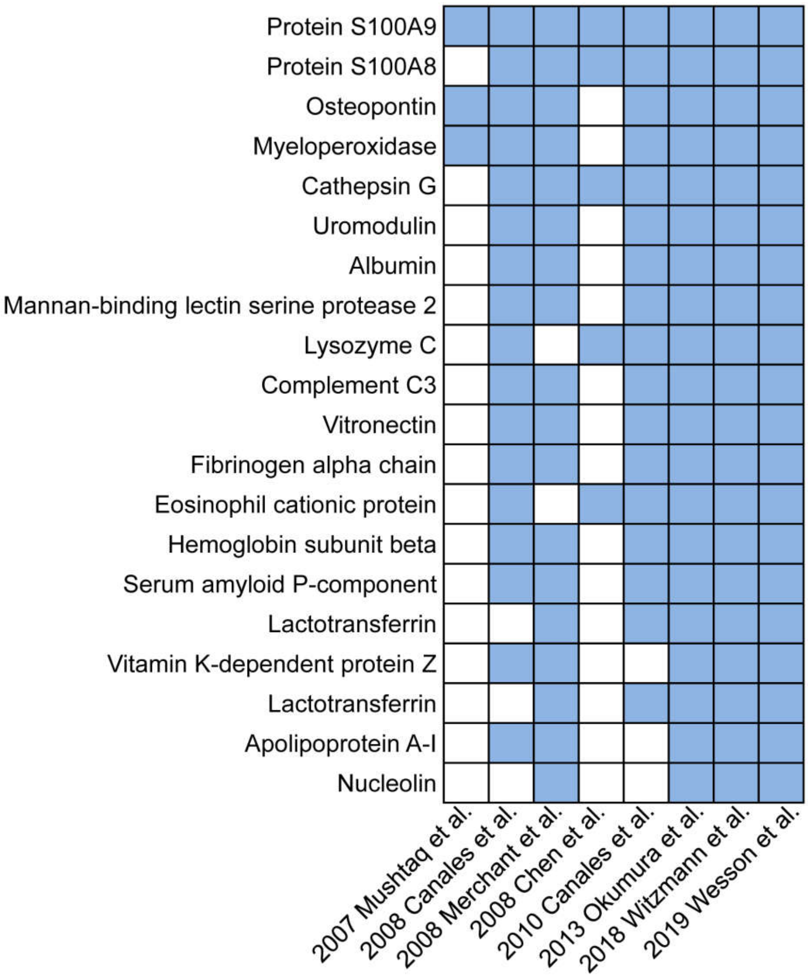

Figure S1. The 20 most common proteins in calcium oxalate stone matrix.
